# Teaching an old dog new tricks: The plant-specific role of VPS41 in vacuolar transport and development

**DOI:** 10.1093/plphys/kiac186

**Published:** 2022-04-23

**Authors:** Yana Kazachkova

**Affiliations:** Department of Plant and Environmental Sciences, Weizmann Institute of Science, Rehovot 7610001, Israel

The vacuole is the largest organelle in plant cells and plays a role in specialized metabolite accumulation, ion homeostasis, protein storage, degradation, and recycling. In plants, two major types of vacuoles exist. Most vegetative cells contain one large acidic lytic vacuole that is similar to the lysosome in animal cells. Protein storage vacuoles are seed specific and accumulate substantial amounts of storage proteins during seed development (Shimada et al., 2018).

How do metabolites and proteins reach the vacuole? Membrane trafficking is crucial for transporting proteins, lipids and metabolites. A single event of membrane trafficking consists of four sequential steps: (1) the formation of the vesicle from a donor membrane; (2) transport of the vesicle; (3) tethering of the vesicle to the target membrane; and (4) fusion of the cargo-laden vesicle to the destination organelle membrane (Minamino and Ueda, 2019). Four major groups of proteins are necessary for correct vesicle fusion: Ras-associated binding guanine triphosphatases, soluble *N*-ethylmaleimide-sensitive fusion protein attachment receptor), tethering protein complexes, and accessory proteins (Cruz and Kim, 2019).

In yeast, the homotypic fusion and vacuole protein sorting (HOPS) complex is a major tether, containing six vacuolar protein sorting (VPS) subunits. HOPS mediates membrane tethering and vacuole fusion (Cruz and Kim, 2019). In plants, single copies of VPS subunit genes exist. VPS41 plays a role in pollen fertility and vacuole fusion in Arabidopsis (*Arabidopsis thaliana*) seedlings (Lihong et al., 2016; Brillada et al., 2018)*.* In pollen tubes, VPS41 protein is associated with prevacuolar compartments and the tonoplast and is necessary for pollen tube–stigma interaction (Lihong et al., 2016). *vps41* mutants have severely fragmented vacuoles, indicating that VPS41 is necessary for correct vacuole fusion (Brillada et al., 2018).

In this issue of *Plant Physiology*, Jiang et al. (2022) investigated the function of VPS41 in vegetative tissues. In agreement with previous data, the researchers showed that in root meristem cells VPS41–GFP fusion protein localizes not only to the tonoplast membrane, but also to punctate structures in the cytosol. To investigate the nature of these puncta, the authors first compared their distribution with the known early and late endosomal proteins and organelle markers. No significant substantial colocalization was observed with any of these markers, with the exception of the tonoplast and prevacuole marker VHA-a3 (a subunit of vacuolar H^+^-ATPase). Immunolabeling using anti-VPS41 antibodies further indicated tonoplast localization of VPS41. However, gold particles also labeled electron-dense structures associated with the endoplasmic reticulum and tonoplast membrane or localized in the cytosol. These structures had no delimiting membrane, indicating that VPS41 molecules in the cytosol might localize to the membraneless compartments that exhibit liquid-like properties. Treatment with 1,6-hexanediol, known to disrupt liquid-like structures, caused complete loss of VPS41 puncta from the cytosol. These data indicate that in root meristem cells VPS41 exhibits dual localization: tonoplast and unknown structures with liquid-like properties.

**Figure 1:**
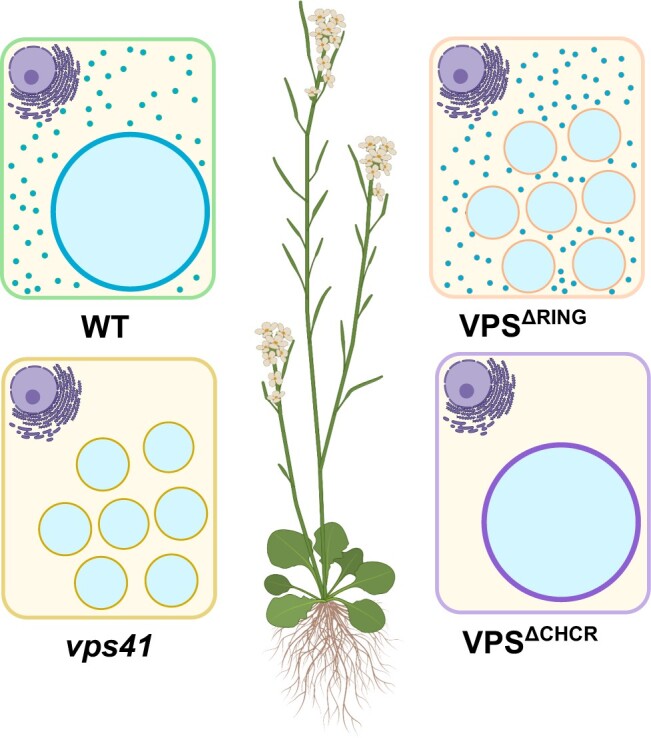
Schematic representation of root meristem cells in wild-type (WT) Arabidopsis plants and *VPS41* mutant lines. Intact or fragmented large lytic vacuoles are shown. Punctate structures are indicated by blue dots.

In yeasts and mammalian cells, VPS41 homologs participate in direct Golgi-to-vacuole transport. Interaction occurs with the adaptor protein complex subunit, AP-3, that links cargo with the vesicle coat proteins (Zwiewka et al., 2011). VPS41 can also be recruited to the endosomes after activation of Rab7, a GTPase that has a role in vesicle formation, transport and fusion (Solinger and Spang, 2013). Using crosses of VPS41–GFP plants with *rab7* or *ap3* Arabidopsis mutants, the authors showed that the plants still exhibited punctate structures in the root cells. Moreover, in *rab7*, VPS41 was depleted from the tonoplast, but a dramatic significant increase in the number of punctate structures in the cytosol was observed (Figure 1). These data indicate that VPS41 puncta do not rely on AP-3 or Rab7 pathways; however, Rab7 is indispensable for correct localization of VPS41 to the tonoplast membrane.

Next, the authors investigated VPS41 functions as a part of the HOPS complex and independent of the complex. In pollen-rescued *vps41* mutant seeds, protein storage vacuoles were severely fragmented and processing of one seed storage protein was impaired. This indicates that VPS41 as a part of the HOPS tethering complex is necessary for cargo sorting and vacuole fusion in plants, similar to mammalian and yeast cells (Asensio et al., 2013). To investigate VPS41 functions independent of HOPS, the RING domain, normally responsible for integration of VPS41 into the HOPS complex, was deleted from the VPS41 protein. In Arabidopsis plants harboring a truncated version of VPS41 protein (VPS41^ΔRING^), the presence of VPS41 on the tonoplast membrane was reduced, but the number of punctate structures in the cytosol remained unchanged. Vacuoles in these plants were severely fragmented, indicating that VPS41 as a part of the HOPS complex is necessary for vacuole fusion but not for the presence of punctate structures in the cytosol. However, the known cargo proteins of Rab5, Rab7, and Golgi-independent trafficking pathways were nevertheless delivered to the fragmented vacuole. This evidence indicates that VPS41 has distinct roles in correct vacuole fusion as a part of the HOPS complex and also carries out transport functions independent of HOPS.

The researchers also investigated how the puncta formed in the cells. Previous reports indicated that in yeast and mammalian cells, VPS41 contains the C-Terminal Clathrin Heavy-Chain Repeat (CHCR) domain that is responsible for self-interaction of VPS41 protein (Asensio et al., 2013). Transgenic lines harboring truncated VPS41 protein lacking the CHCR domain (VPS41^ΔCHCR^) had significantly fewer punctate structures in the cytosol and exhibited severe growth defects. This suggests that VPS41 has important roles in plant vegetative development independent of the HOPS protein complex.

In summary, Jiang et al. showed that in addition to its role in vacuole fusion as a part of the HOPS complex, VPS41 has further distinct functions in vacuolar transport regulation. However, the exact role of VPS41 in puncta as well as its HOPS-independent mechanisms in root meristem cells remain elusive. This study complements the evidence that in addition to conserved mechanisms of vacuole trafficking among eukaryotes, plants possess unique transport pathways that appeared during the course of evolution.

*Conflict of interest statement*. None declared.
